# The contribution of prognostic factors to socio‐demographic inequalities in breast cancer survival in Victoria, Australia

**DOI:** 10.1002/cam4.6092

**Published:** 2023-07-17

**Authors:** Geoffrey W. Stuart, James A. Chamberlain, Luc te Marvelde

**Affiliations:** ^1^ Cancer Epidemiology Division Cancer Council Victoria Melbourne Victoria Australia; ^2^ School of Psychological Sciences, Faculty of Medicine, Dentistry and Health Sciences University of Melbourne Victoria Melbourne Australia; ^3^ Victorian Cancer Registry Cancer Council Victoria Melbourne Victoria Australia

**Keywords:** Australia, breast cancer, health disparities, prognostic factors, rural health, socio‐economic status, survival

## Abstract

**Background:**

Breast cancer survival in Australia varies according to socio‐economic status (SES) and between rural and urban places of residence. Part of this disparity may be due to differences in prognostic factors at the time of diagnosis.

**Methods:**

Women with invasive breast cancer diagnosed from 2008 until 2012 (*n* = 14,165) were identified from the Victorian Cancer Registry and followed up for 5 years, with death from breast cancer or other causes recorded. A prognostic score, based on stage at diagnosis, cancer grade, whether the cancer was detected via screening, reported comorbidities and age at diagnosis, was constructed for use in a mediation analysis.

**Results:**

Five‐year breast cancer mortality for women with breast cancer who were in the lowest quintile of SES (10.3%) was almost double that of those in the highest quintile (5.7%). There was a small survival advantage (1.7% on average, within each socio‐economic quintile) of living in inner‐regional areas compared with major cities. About half of the socio‐economic disparity was mediated by prognostic factors, particularly stage at diagnosis and the presence of comorbidities. The inner‐regional survival advantage was not due to differences in prognostic factors.

**Conclusions:**

Part of the socio‐economic disparity in breast cancer survival could be addressed by earlier detection in, and improved general health for, more disadvantaged women. Further research is required to identify additional causes of socio‐economic disparities as well as the observed inner‐regional survival advantage.

## Graphical abstract

Five‐year breast cancer mortality for women in Victoria, Australia who were in the lowest quintile of socio‐economic status was almost double that of those in the highest quintile. There was a small survival advantage of living in inner‐regional areas compared with major cities. About half of the socio‐economic disparity was mediated by prognostic factors, particularly stage at diagnosis and the presence of comorbidities. The inner‐regional survival advantage was not due to differences in prognostic factors.

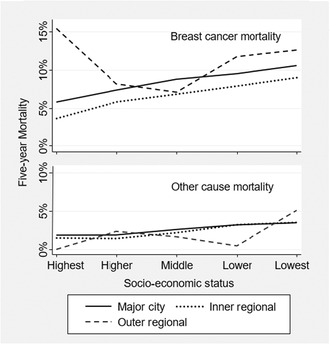

## 1 INTRODUCTION

Many epidemiological studies have reported survival disparities for various cancers, across a range of countries, as a function of socio‐economic status (SES).^1^, ^2^ Less consistently, it has been found that cancer patients who reside outside major urban areas, and especially those in more remote rural areas, may also have higher cancer mortality, even after adjusting for SES.^3^, ^4^ These socio‐demographic disparities represent preventable deaths. In Australia these inequalities are recognised in official policy as unacceptable health outcomes.^5^ While survival from breast cancer specifically has progressively improved in Australia,^6^ socio‐economic disparities have widened^7^, ^8^ and there have been reports that rural–urban disparities are also widening.^9^ To address these inequalities, it is necessary to identify their causes. The present study is concerned with prognostic factors that may place breast cancer patients of low SES, or those who are living in rural areas, at greater risk of dying from their disease.

Internationally, a range of reviews and meta‐analyses have identified socio‐economic disparities in breast cancer survival^1^, ^10^, ^11^, ^12^, ^13^ and studies continue to be published.^14^, ^15^, ^16^, ^17^ These studies consistently demonstrate that survival rates are lower in more economically disadvantaged groups. Disparities associated with rural versus urban residence are not consistent in the case of breast cancer.^4^ Outcomes vary across countries with rural residence sometimes associated with lower risk of mortality. With a few exceptions^18^, ^19^, ^20^, ^21^, ^22^, ^23^ typical studies have estimated rural–urban disparities after adjusting for various socio‐economic factors.^24^, ^25^, ^26^, ^27^, ^28^ This is an important consideration, because it is possible that rural–urban survival disparities are in part due to factors such as distance to health services or the quality of those services.

There have been several Australian studies that have examined socio‐economic and rural–urban disparities in breast cancer mortality.^29^, ^30^ Some have examined socio‐economic disparities alone^7^, ^31^ rural–urban disparities alone^24^, ^26^ or both.^8^, ^9^, ^32^, ^33^, ^34^ In most Australian studies that included a measure of cancer stage at initial diagnosis, the effect of stage on survival was either statistically controlled when estimating socio‐economic and rural–urban disparities^7^, ^8^, ^9^, ^32^, ^34^ or it was used to stratify the sample, and then disparities were examined within stage.^26^ This has the advantage of isolating the potential contribution of other factors, such as treatment. However, it means that the contribution of stage to survival disparities was not quantified, and the strength of this contribution is important to inform policy and practice to reduce survival disparities.

The typical approach when examining the role of prognostic or treatment‐related factors in relation to cancer survival disparities uses regression methodology.^28^, ^35^, ^36^, ^37^, ^38^, ^39^, ^40^ The mediating effect of these factors on survival is assessed by noting the change in the regression coefficient for SES (or rurality) when these variables are added to the regression model. While a significant change in regression coefficients represents evidence for some mediating effect, the change in coefficients is not an accurate measure of the strength of that effect.^41^, ^42^ In addition, standard regression methods are also subject to bias from various types of confounding. For example, SES may affect the stage of cancer at diagnosis, which in turn affects survival. However, if patients of different SES do not receive the same treatment given the same stage of the disease, this may contaminate estimates of the mediating effect of stage of diagnosis between SES and survival. More sophisticated methods that fall under the rubric of causal mediation analysis^43^, ^44^ are required to deal with this type of confounding. Mediation analysis has previously been applied to the question of socio‐economic disparities in breast cancer survival.^45^, ^46^ Only one prognostic factor (stage at diagnosis) and one treatment factor (time to surgery) were considered. The treatment measure did not strongly mediate survival disparities, but stage at diagnosis accounted for a third of the socio‐economic disparity.

In the present study we conducted a more in‐depth analysis of the role of multiple prognostic factors, including stage at diagnosis, in mediating breast cancer‐specific survival at 5 years post‐diagnosis. We analysed how the prognostic factors mediated disparities in the survival outcome by SES and rurality, using data from the Victorian Cancer Registry (VCR) linked to hospital and death records.

## 2 METHODS

### 2.1 Data sources

Breast cancer patient data were drawn from the VCR, to which the details of all pathology‐confirmed cancers diagnosed in Victoria, Australia (other than minor skin cancers) are reported. The VCR collects data on patient demographics and tumour details (site, morphology, grade, behaviour, date of diagnosis). The Victorian Admitted Episodes Dataset (VAED) includes demographic and clinical data for in‐patient admissions to any public or private Victorian hospital, including rehabilitation centres, extended care facilities and day procedure centres. Clinical diagnosis data are recorded using the International Statistical Classification of Diseases and Related Health Problems, 10th Revision, Australian Modification (ICD‐10‐AM). The VCR is linked monthly to the Victorian Registry of Births, Deaths and Marriages, and annually to the National Death Index at the Australian Institute of Health and Welfare, to update vital status and cause of death. Cause of death is coded by trained medical coders from the VCR using text description from the actual death certificate, WHO underlying cause of death rules and additional information known about the patient. Data linkage between the VCR and VAED was performed by the Centre for Victorian Data Linkage using an iterative deterministic linkage process with fuzzy matching on selected fields that enabled the matching of patients in the two data sets.

### 2.2 Inclusion and exclusion criteria

The sample initially included 15,423 women diagnosed with invasive breast cancer (ICD‐10 codes C500–C509) between 2008 and 2012, who were aged 78 years or under, were not notified by death certificate only and were followed up until the end of 2017. The age exclusion was applied because treatment options and prognosis differ for older breast cancer patients. Cases notified by death certificate only were excluded. Also excluded were cases with unknown stage or stage measured after neoadjuvant therapy (*n* = 1202) and 56 cases whose socio‐demographic variables could not be linked by area of residence. This left 14,165 cases for analysis.

Cause of death, whether from breast cancer or another cause (including other cancers) was determined by the VCR according to a detailed protocol that considered a range of sources of information, including multiple causes listed on death certificates, hospital records and information from the cancer registry.^47^

### 2.3 Prognostic factors

Three factors in the VCR dataset that are directly or indirectly related to tumour characteristics at the time of diagnosis were considered, namely summary stage, grade and whether the cancer was detected via the state screening mammography program. Summary stage was determined from pathology reports using the tumour, node and metastases (TNM) staging system.^48^ Cancer grade was also determined from pathology reports. A substantial proportion of cases, particularly those diagnosed with stage 4 disease, did not have their cancer graded. A separate category of grade unknown was created for these women so that they could be retained in the broader analysis. Screen detection was determined through record linkage to the records of BreastScreen Victoria, which forms part of Australia's comprehensive, free breast cancer screening program. These records do not include cancers detected through private screening services, which have been previously estimated to be used by an additional 4%–5% of women in the age range targeted by the public BreastScreen program in Australia.^49^

Additional prognostic factors not directly related to tumour characteristics were age at diagnosis and the presence of comorbidities. The presence of comorbidities was derived from VAED records and depended on whether comorbidities were noted in relation to hospital admissions in the year prior, or 30 days after, breast cancer diagnosis. The Charlson Comorbidity Index,^50^ excluding cancer, was calculated for each patient. Comorbidity weights were applied according to Quan et al.^51^ and grouped as zero or at least one comorbidity. For many cases there was no hospital admission in the defined period around diagnosis, and a separate category of ‘not reported’ was created for the purpose of statistical analysis.

### 2.4 Socio‐demographic indicators

Socio‐demographic indicators were derived from Australian Bureau of Statistics (ABS) 2011 Census data for the smallest geographical areas (SA1). There are nearly 13,000 such areas in the state of Victoria, each with an average population of approximately 400 people. These area‐based indicators were linked to individual patient records by geo‐coding residential addresses at the time of cancer diagnosis.

#### 2.4.1 Socio‐economic status

The ABS Socio‐Economic Indexes for Areas (SEIFA) comprises four indices which rank areas of Australia according to socio‐economic advantage and disadvantage, using socio‐economic variables from the 2011 census. We used the SEIFA Index of Relative Socio‐Economic Disadvantage as the measure of SES.^52^ When referring to SES, low SES, therefore, corresponds to a high level of socio‐economic disadvantage. and vice‐versa.

#### 2.4.2 Rurality

The ABS assigns a categorical ‘remoteness’ index to each statistical area (SA1) used in the Australian census, based on the Accessibility/Remoteness Index of Australia (ARIA+). The continuous index is reduced to five categories by the ABS, namely ‘Major Cities’, ‘Inner Regional’, ‘Outer Regional’, ‘Remote’ and ‘Very Remote’.^53^ The state of Victoria has no very remote areas and a very small proportion of remote areas. For analysis purposes the remote areas were merged with the outer regional areas, yielding three categories of rurality.

### 2.5 Statistical analysis

#### 2.5.1 Data visualisation

In addition to standard line plots of means and percentages, mosaic plots^54^ were used to illustrate the relationships between categorical variables.

#### 2.5.2 Multinomial logistic regression

Since there were three possible outcomes in the 5 years following breast cancer diagnosis (survival, death from breast cancer or death from other causes), initial multivariate analyses used multinomial logistic regression to estimate the effect of SES, rurality and prognostic factors on competing causes of death.

#### 2.5.3 Binary Poisson regression

The relationship between prognostic factors and the probability of dying from breast cancer compared with surviving was explored in further detail using Poisson regression with robust variance estimation.^55^ The method provides easily interpretable, fully adjusted risk ratios when combined with offset‐based regression, described below.

#### 2.5.4 Regression using offsets

Multiple regression coefficients, while optimised for statistical prediction, cannot be unambiguously interpreted as measures of individual variable importance when predictors are correlated.^56^, ^57^ To isolate the direct contribution of individual prognostic factors to survival, the technique of regression with offsets^58^ was used. In this method, for each independent variable in a set, all other variables are first used in a multiple regression. The regression coefficients are then fixed, and the remaining variable is added to the regression model. The resulting regression coefficient (and the associated risk ratio) represents the unique, fully controlled contribution of the selected variable to the outcome.

#### 2.5.5 Prognostic scores

Complex relationships between multiple prognostic factors present a challenge to understanding how these prognostic factors mediate the observed relationship between SES, rurality and survival. Prognostic scores (also known as disease risk scores when the outcome is incidence of disease)^59^ were used as a single continuous mediator between the exposures (SES and rurality) and survival. A theoretical challenge with this method is the problem of exposure‐induced mediator‐outcome confounding^60^ as described above. To eliminate this confounding, prognostic scores were based on the prognostic risk in a reference group (high SES city dwellers). This means that the prognostic score in the other groups is not affected by unmeasured confounding from factors that differentially affect those groups post‐diagnosis, such as treatment (see Supplementary Material for a more detailed explanation).

#### 2.5.6 Mediation of survival disparities due to differential prognosis

To estimate the amount of survival disparity due to prognostic factors, the expected rates of survival across the relevant SES or rurality groups were calculated solely on the basis of prognostic scores. These expected rates were then compared to the actual rates. The discrepancy between these rates was quantified as percentage mediated, or as differences between observed disparities and those expected from differential prognosis, as a function of SES and rurality. Confidence intervals (CIs) were based on bias‐corrected bootstrap estimates.^61^

## 3 RESULTS

### 3.1 Inequalities in survival

**FIGURE 1:**
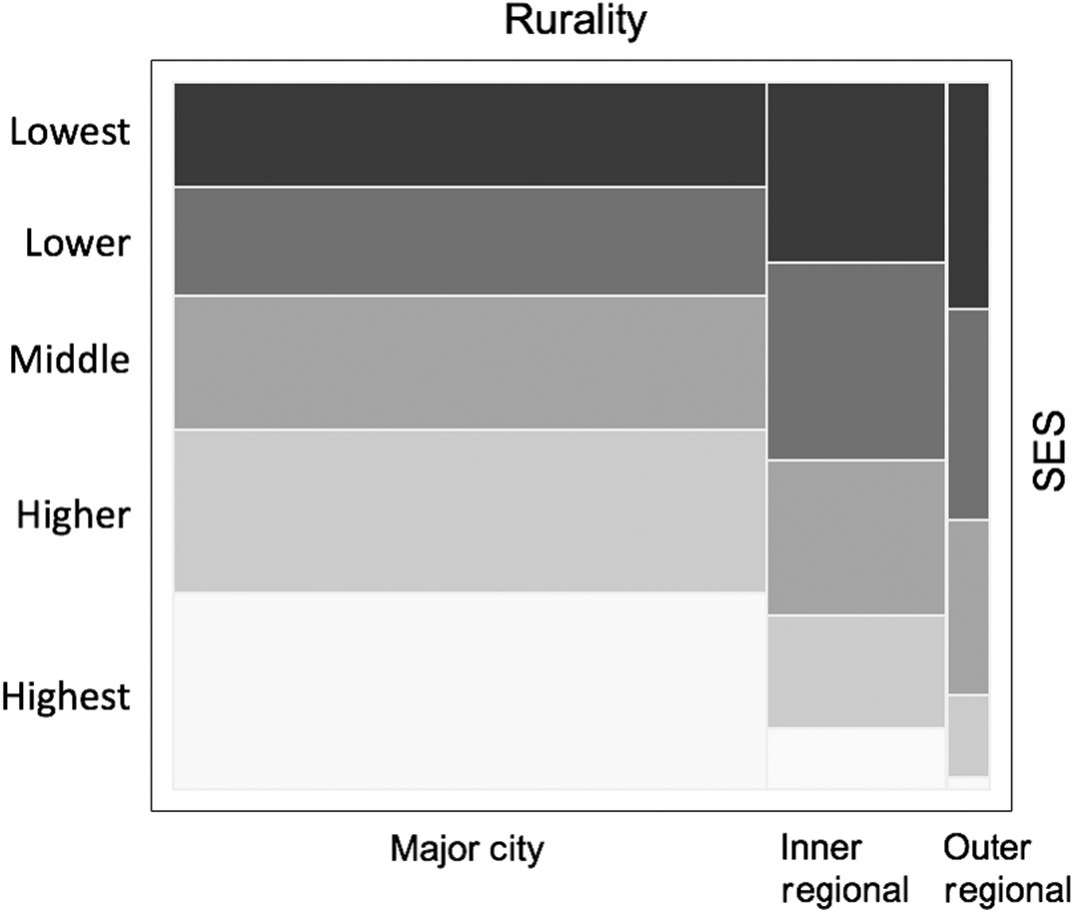
Rurality and socio‐economic status (SES) in the breast cancer cohort. The size of the ‘tiles’ in the mosaic plot represent the proportion of the cohort falling into each category of SES and rurality.

There was an association between SES and rurality based on area of residence (Figure 1), with an increasing proportion of areas of lower SES with increasing rurality.

**FIGURE 2:**
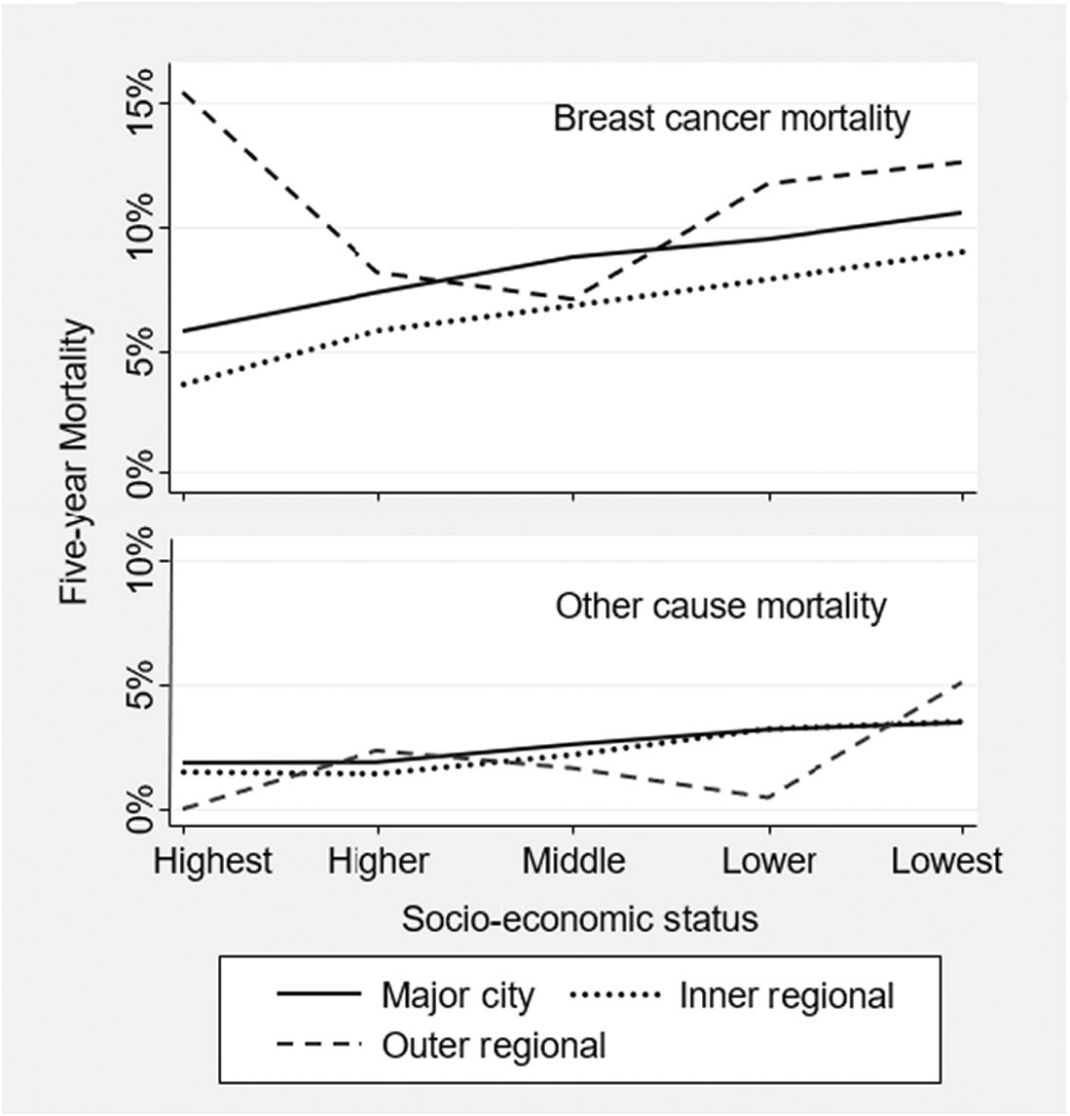
Five‐year breast cancer‐specific and other‐cause mortality as a function of socio‐economic status and rurality of the statistical area of residence. Points on the plot were derived from widely varying numbers of cases (see Figure 1).

Figure 2 shows the breast cancer mortality and other‐cause mortality as a function of SES and rurality. Exact percentages and unadjusted CIs are given in Supplementary Material. There were no statistically significant interactions on the logit scale, with a maximum interaction odds ratio of 1.03 (95% CI = 0.91–1.17). Additive multinomial logistic regression was then used to model associations of SES and rurality with both breast cancer mortality and other‐cause mortality, as illustrated in Figure 2. Using cases who survived as the base category there was a pronounced increase in the probability of breast cancer mortality with increasing socio‐economic disadvantage (odds ratio [OR] = 1.19 per quintile, 95% CI = 1.13–1.24, *p* < 0.001) representing a doubling of mortality across the range. There was also a survival benefit associated with inner regional relative to metropolitan residence that was consistent across SES categories (OR = 0.80, 95% CI = 0.68–0.94). Other‐cause mortality was relatively low, approximately 2%–3% across SES categories, with a similar increase in relative odds of other‐cause death with increasing disadvantage (OR = 1.24 per quintile, 95% CI = 1.15–1.34, *p* < 0.001), but no indication of an inner‐regional benefit (OR = 0.92, 95% CI = 0.71–1.19).

Due to the sparsity of data in the higher SES categories within outer regional areas (Figure 1), we conducted secondary analyses of rurality using only the three lower SES categories. There was significantly higher breast cancer mortality in outer regional areas compared to inner‐regional areas (OR = 1.35, 95% CI = 1.02–1.82, *p* = 0.039), but there was no significant difference in mortality between outer regional and major city areas (OR = 1.11, 95% CI = 0.85–1.45, *p* = 0.45).

### 3.2 Prognostic factors

**FIGURE 3:**
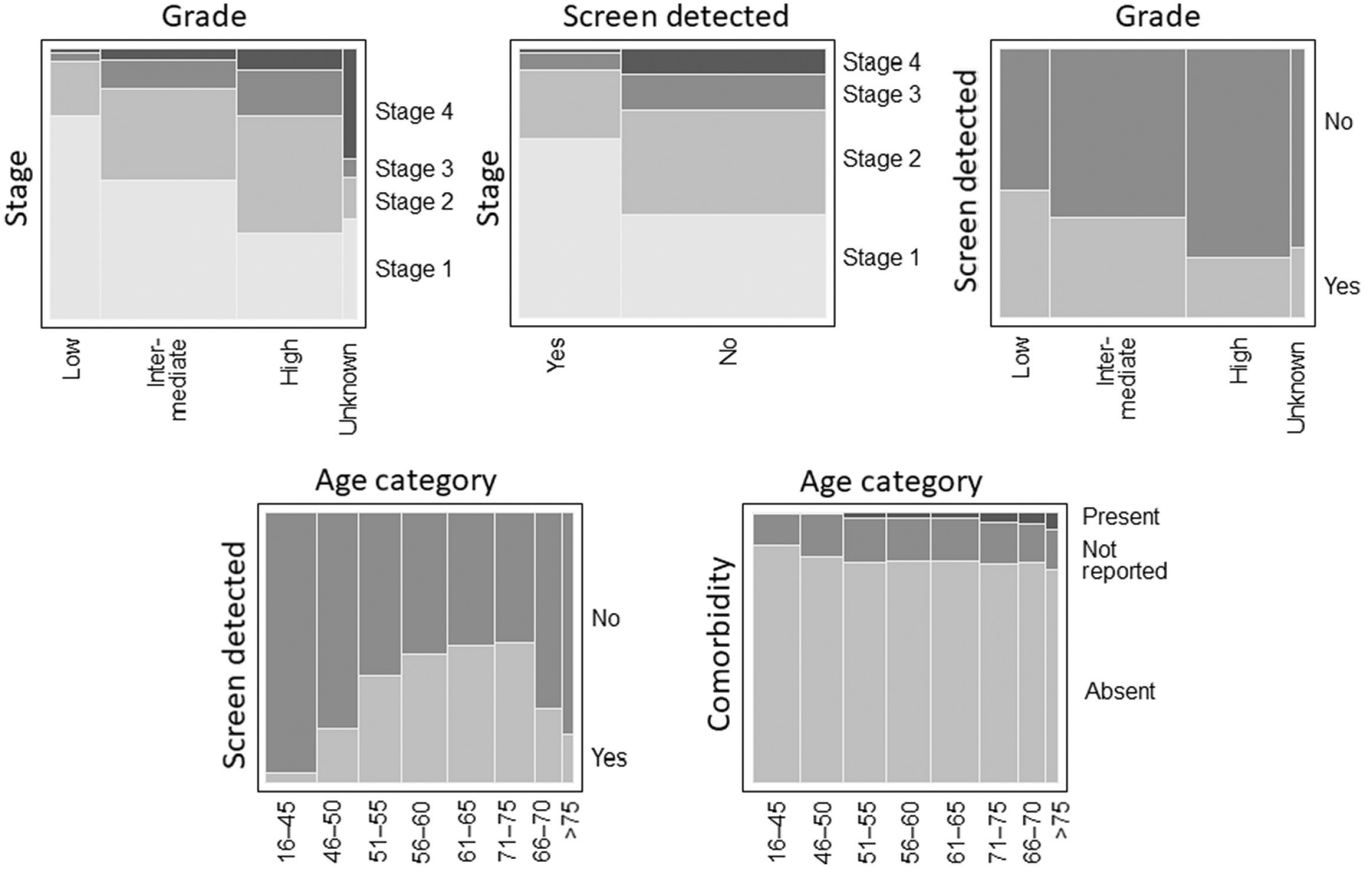
Pairwise relationships between selected prognostic factors.

Prior to multivariate analysis, pairwise associations between the prognostic factors were examined. These associations were complex. As shown in Figure 3, screen detection was more likely within the age range of population‐based screening programs, cancers detected via screening were diagnosed at an earlier stage, and stage at diagnosis decreased with age up until the age of 65 years and then increased. Higher‐grade tumours were less likely to be screen detected and also tended to be diagnosed at a later stage. Stage 4 tumours tended to have a higher proportion of unknown grade, and comorbidity increased with age. Other associations were weaker (see Supplementary Material).

**FIGURE 4:**
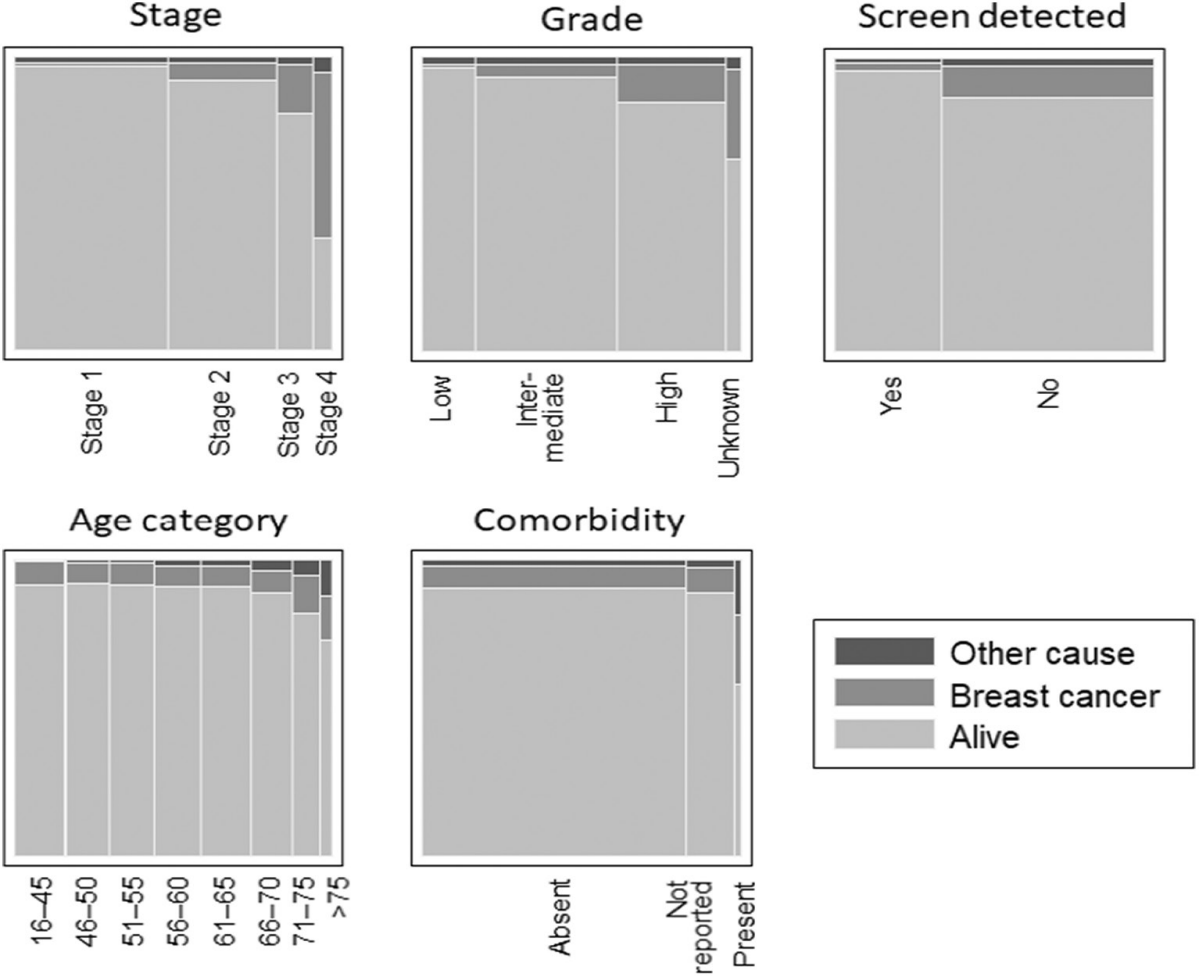
Relationship between individual prognostic factors and breast cancer‐specific and other‐cause mortality.

Figure 4 illustrates how each prognostic factor, treated individually, related to breast cancer mortality and other‐cause mortality. Multinomial logistic regression was used to examine the combined influence of these prognostic factors on five‐year breast cancer and other‐cause mortality. That analysis indicated that all prognostic factors contributed to the prediction of breast cancer mortality and some to other‐cause mortality. Details are provided in the Supplementary Material. We note here only that (i) other‐cause mortality was very low and (ii) women who died from other causes tended to be older, to have other illnesses and to have later stage cancer than survivors, even though they did not die from cancer. To gain a fuller picture of the relationship between prognostic factors and breast cancer mortality specifically, including the inter‐relationships between prognostic factors, we conducted a more detailed analysis comparing those women who died from breast cancer to those who survived for 5 years (excluding other‐cause mortality), using binary Poisson regression.

There were no interaction terms that improved the fit of a standard logistic regression model (see Supplementary Material for details) and so subsequent analyses used additive models. Table 1 shows univariate, multiple regression and partial (offset) risk ratios for prognostic factors for breast cancer‐specific mortality, all based on Poisson regression. To aid interpretation, note for example that unadjusted risk ratios associated with unknown and high grade were very different, but the multiple regression and partial (offset) risks ratios were similar. However, there was a greater frequency of stage 4 cancers with unknown grade (Figure 3). This explains why, when stage was included in the models, the risk ratios for grades ‘High’ and ‘Unknown’ were similar.

**TABLE 1:** Univariate, multiple regression‐based and partial (offset) risk ratios associated with different categories of prognostic risks for breast cancer‐specific mortality.

	*N* (%)	Raw mortality rate (%)	Univariate risk ratio	95% Confidence interval	Multiple regression risk ratio	95% Confidence interval	Partial (offset) risk ratio	95% Confidence interval
Stage								
Stage 1	6823 (48.2)	1.15						
Stage 2	4856 (34.3)	5.90	5.12	3.99–6.57	3.64	2.82–4.69	3.20	2.50–4.11
Stage 3	1584 (11.2)	16.89	14.66	11.44–18.78	9.44	7.30–12.22	7.88	6.15–10.09
Stage 4	902 (6.4)	59.98	52.04	41.40–65.41	27.83	21.74–35.63	14.37	11.38–18.14
Grade								
Low	2351 (16.6)	1.00						
Intermediate	6302 (44.5)	4.36	4.35	2.85–6.64	2.30	1.54–3.43	2.14	1.45–3.16
High	4849 (34.2)	13.43	13.40	8.86–20.25	4.84	3.26–7.12	4.33	2.96–6.34
Unknown	663 (4.7)	31.60	31.52	20.66–48.09	4.39	2.93–6.59	3.72	2.53–5.46
Comorbidity								
None	11,735 (82.9)	7.62						
Not reported	2136 (15.1)	8.78	1.15	0.99–1.34	1.16	0.98–1.27	1.11	0.98–1.27
Present	294 (2.1)	28.87	3.79	3.07–4.67	1.52	1.30–1.79	1.50	1.28–1.76
Screen detected								
Yes	4756 (33.6)	2.27						
No	9409 (66.4)	11.01	4.28	3.55–5.15	1.71	1.43–2.05	1.60	1.35–1.90
Age (years)								
16–45	2276 (16.1)	7.74						
46–50	1947 (13.8)	7.09	0.92	0.74–1.14	1.16	0.96–1.41	1.15	0.95–1.40
51–55	2001 (14.1)	7.33	0.95	0.77–1.17	1.26	1.05–1.52	1.24	1.03–1.49
56–60	2093 (14.8)	6.93	0.89	0.72–1.11	1.27	1.05–1.53	1.24	1.04–1.50
61–65	2209 (15.6)	6.81	0.88	0.71–1.09	1.26	1.05–1.51	1.23	1.03–1.48
66–70	1824 (12.9)	7.80	1.01	0.81–1.25	1.52	1.25–1.84	1.48	1.23–1.79
71–75	1250 (8.8)	13.65	1.76	1.44–2.16	1.86	1.56–2.22	1.82	1.53–2.16
>75	565 (4.0)	16.73	2.16	1.70–2.75	2.10	1.73–2.56	2.04	1.68–2.49

As shown in Table 1, the relative importance of different prognostic factors in determining breast cancer mortality was quite consistent across all three risk estimates, although as expected, fully partialled estimates of relative risk were much lower than the unadjusted estimates. Stage was most strongly associated with breast cancer mortality, followed by grade. Comorbidity, and whether the cancer was detected though screening, also affected breast cancer mortality, but to a lesser degree. Even after fully adjusting for all other factors (offset risk ratios in Table 1), factors other than stage influenced breast cancer survival. For example, the risk ratios were 1.50 for presence of comorbidities, 1.60 for non‐screen detected cancer and 4.33 the grade 4 relative to grade 1 disease.

### 3.3 Prognostic score as a mediator of socio‐demographic inequalities in survival

**FIGURE 5:**
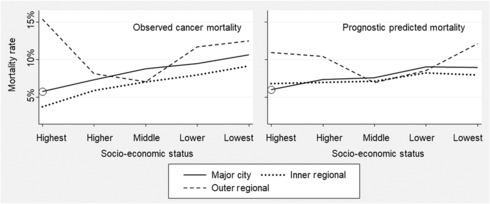
Observed rates of cancer‐specific mortality compared to predicted mortality rates based on prognostic scores. Circles represent the reference group (highest socio‐economic status, major city) used for estimating prognostic scores.

Figure 5 shows expected breast cancer mortality based on prognostic score alone (right panel) in categories defined by SES and rurality, compared with the corresponding observed breast cancer mortality (left panel). Residents of lower SES areas were at greater risk of dying from breast cancer due to the prognostic factors considered, and the change in risk across SES categories was approximately linear. Table 2 shows estimates of the extent to which differences in prognostic factors collectively explain observed differences in breast cancer‐specific mortality, according to SES and rurality of residence (inner regional vs. metropolitan only). An estimated 49.7% of the increased breast cancer mortality due to lower SES could be accounted for by prognostic score. In contrast, the prognostic benefit associated with inner‐regional residence was small relative to the observed benefit, at around a quarter of the observed benefit, and the 95% bootstrap CI for the predicted inner‐regional benefit due to prognosis included zero.

**TABLE 2:** Prognostic score‐predicted and observed 5‐year breast cancer‐specific mortality as a function of SES and rurality of residence.

Measure	Point estimate (%)	Bias‐corrected bootstrap 95% confidence interval	
		Lower (2.5 %)	Upper (97.5%)
Increased observed mortality per SES quintile	1.26	0.91	1.60
Increased prognostic score per SES quintile	0.62	0.45	0.78
Percentage mediated for SES due to prognostic factors	49.7	39.2	66.2
Observed inner‐regional versus metro mortality benefit	1.66	0.57	2.76
Prognostic inner‐regional versus metro mortality benefit^a^	0.42	−0.07	0.91
Difference b/w observed and prognostic benefit	1.24	0.33	2.19

Abbreviation: SES, socio‐economic status.

^a^ This corresponds to 25.3% of the inner‐regional benefit being mediated by prognostic factors. The confidence interval for the difference between and observed and predicted prognostic benefit includes a Metro mortality benefit, and so the confidence interval for the percentage mediated is undefined (negative mediation is not possible).

Stage dominated the prognostic score, as shown by the high risk ratios in Table 1. Using pseudo‐*R* ^2^ measures of binary model fit, a prognostic model using stage alone accounted for 86% of the variance in survival explained by the full prognostic model. However, the other factors all significantly improved the performance of the prognostic model.

## 4 DISCUSSION

In this population‐based study we followed up 14,165 women who were diagnosed with breast cancer between 2008 and 2012, for 5 years. The main outcome was breast cancer‐specific survival at 5 years from the date of diagnosis. We found that approximately half the observed socio‐economic disparity in breast cancer‐specific survival was associated with the prognostic factors considered, namely age, stage at diagnosis, cancer grade, whether the cancer was detected via mammographic screening and the presence of comorbidities. Only around a quarter of the lower breast cancer mortality in inner‐regional areas was associated with those factors but that proportion was not statistically significant. As expected, the relationship between the prognostic factors themselves and the socio‐economic and urban/rural measures of interest was complex. We, therefore, developed a prognostic score to capture the combined mediating effect, while adjusting for any unmeasured confounding that might arise from post‐diagnostic factors such as treatment.

Given the non‐independence of the prognostic factors, the overall prognostic score was designed to capture all the variance in those factors, both shared and unique, that contributed to the probability of survival from breast cancer and that mediated the effects of SES and rurality on survival. We also examined the unadjusted and unique (fully adjusted) effects of each prognostic factor on survival. This showed that each prognostic factor made at least some contribution to survival independently of the others. Consistent with previous research^62^, ^63^, ^64^ we found that stage at diagnosis was an important component of the prognostic model.

A strength of the present study is the population‐based design and large sample size. We controlled for mediator‐outcome confounding due to any effects of socio‐economic and rural–urban status on unmeasured post‐diagnosis factors, such as treatment; neglecting this confounding could have led to overestimation of the effect of prognostic factors such as stage at diagnosis. However, there were several limitations to the study. In common with most studies using cancer registry data, we used area‐based measures of SES derived from residential address, not individual‐level measures such as income. This may have attenuated the strength of the relationship between SES and survival.^17^ Although the comorbidity measure used was strongly associated with survival in lung cancer patients among various jurisdictions including Victoria,^65^ the measure used was not comprehensive, relies on reporting of comorbidities in administrative data and has not been tested previously in breast cancer patients. Better measurement of comorbid conditions, as well as the inclusion of lifestyle factors such as obesity and smoking, might increase the proportion of survival disparity explained. Our measure of screen detection did not include cancers detected through private screening, only those notified by the public program. We did not include measures of treatment or other factors (such as cultural barriers to optimal health behaviours) that might account for the disparities that were not explained by the prognostic factors considered.

Screening programs aim to detect cancers at earlier stages to improve prognosis. However, stage and screen detection are not interchangeable prognostic factors, as shown by the finding that screen detection had survival benefits even after adjusting for the effect of stage. Other factors such as self‐diagnosis, help‐seeking in relation to early symptoms, and ease and timeliness of access to health services, may influence stage at diagnosis.^64^ Maly et al.^66^ found that delay between self‐detection of breast abnormalities and definitive diagnosis was influenced by perceived self‐efficacy in interacting with healthcare providers. Conversely, the beneficial effects of earlier detection through screening may not be completely captured by the categorisation into four broad stage categories. There may be information in the individual measures that are used for TNM staging that further mediate the effects of earlier detection on survival. Another possibility is that the residual benefit of screen detection observed in this study is at least partly an artefact of earlier diagnosis per se on survival 5 years post‐diagnosis, the effect known as lead time bias.^67^ In addition, some cancers detected via screening may have led to overdiagnosis^68^, ^69^, ^70^ whereby some cancers detected at early stages may never have led to serious illness or mortality.

Another modifiable prognostic factor that differs according to SES is comorbidity. Even after controlling for all other prognostic factors, the reported presence of comorbidities in hospital admission data was associated with a 50% increased risk of breast cancer death. This is consistent with previous research showing greater levels of comorbid disease in lower SES groups,^71^, ^72^ and a higher risk of breast cancer death for patients with a higher BMI^73^ or with comorbid conditions.^74^

Given the strong contribution of breast cancer stage to our prognostic score, our findings are consistent with previous studies that, with one exception^35^ found that stage at diagnosis made a contribution to socio‐economic disparities in breast cancer survival.^7^, ^36^, ^38^, ^39^, ^40^, ^75^ We have extended that work, which largely depended on regression‐based adjustment, to provide an unbiased estimate of the proportion of excess mortality in lower SES groups that is *mediated* by the prognostic factors considered.

Turning to rural–urban disparities, we observed a survival advantage for inner‐regional areas, which was consistent across SES quintiles but was largely unexplained by the prognostic factors considered. Some Australian studies of female breast cancer survival have reported no strong disparities in breast cancer survival between inner‐regional areas and urban areas^9^, ^24^, ^26^, ^32^ albeit in different Australian states. Yu et al.^34^ reported that for the most recent period covered in their study (2002–2005), after adjusting for SES (as well as age and stage of diagnosis), the estimated relative excess risk of breast cancer death for women living in inner‐regional areas compared with major city dwellers was 0.90, but this survival advantage was not statistically significant, albeit after adjusting for cancer stage.

As noted by Baade et al.,^62^ the effects of rural living may differ across countries, especially as a function of remoteness. In contrast with Yu et al.^9^ and Tervonen et al.^8^ we found no consistent evidence of survival disadvantage to living in outer regional areas, after the generally lower SES of those areas was considered. There were limited data as the majority of Victorians live in urban or inner‐regional areas. Nevertheless, our findings suggest that any disadvantage associated with distance from specialist cancer treatment services that might apply to outer regional areas does not apply to inner‐regional rural areas, which in Victoria are serviced by regional integrated cancer services located in large regional townships.^5^ Further, it may be that there are some disadvantages to living in major cities (relative to inner‐regional areas) that are not captured by the prognostic factors that were included in the present analysis. Relative to inner‐regional dwellers, city dwellers were less likely to have screen detected tumours, had more advanced cancer stage and more (reported) comorbidity, but this did not explain a large proportion, if any, of the urban survival disadvantage relative to inner‐regional rural areas.

One possible difference between inner‐regional and urban areas is that major cities in Victoria are more culturally diverse, with high proportions of immigrants, whereas inner‐regional areas are relatively culturally homogenous with fewer immigrants.^76^ In Australia, women from Arabic countries^77^, ^78^ and the Indian subcontinent^79^ have lower participation in breast cancer screening programs. In Victoria, these women are primarily resident in urban areas.^76^

Our study could not address disparities due to factors that arise after diagnosis, such as access to optimal treatment. It is notable that when breast cancer patients are enrolled in clinical trials, ensuring the same treatment regime, there are almost no survival disparities by rurality observed,^80^ suggesting that in principle any socio‐economic disparities due to variation in treatment could also be addressed by ensuring similar treatment regimens across socio‐demographic groups.

In conclusion, we show that at least half of the socio‐economic disparity in breast cancer‐specific survival at 5 years post‐diagnosis is due to the prognostic factors considered, in particular stage at diagnosis. This justifies ongoing efforts to detect breast cancer earlier in its course and to reduce the level of comorbidities in socio‐economically disadvantaged groups. Further research is required to identify additional causes of socio‐economic disparities as well as the observed inner‐regional survival advantage.

## AUTHOR CONTRIBUTIONS

**Geoffrey W. Stuart:** Conceptualization (lead); formal analysis (lead); investigation (lead); writing – original draft (lead); writing – review and editing (equal). **James A. Chamberlain:** Conceptualization (supporting); formal analysis (supporting); writing – review and editing (equal). **Luc te Marvelde:** Conceptualization (supporting); data curation (equal); writing – review and editing (equal).

## CONFLICT OF INTEREST STATEMENT

The authors report no conflicts of interest.

## ETHICAL APPROVAL STATEMENT

The study was approved by the Cancer Council Victoria Human Research Ethics Committee.

## Supporting information


Data S1.

## Data Availability

Data from the Victorian Cancer Registry and the Victorian Admitted Episodes Dataset was made available to the researchers with approval from the data custodians at the Victorian Department of Health and Human Services and the Cancer Council Victoria Human Research Ethics Committee, under a memorandum of understanding that does not allow these data to be shared with third parties.
