# Supplementary material for: The contribution of prognostic factors to socio‐demographic inequalities in breast cancer survival in Victoria, Australia
Source: Cancer Med. 2023 Jul 17;12(14):15371–83. doi: 10.1002/cam4.6092 (PMC10417162; doi:10.1002/cam4.6092)
Supplement: Supplementary file 1 — Data S1. [file CAM4-12-15371-s001.docx]

**Online Supplementary Material**

*1. Multinomial logistic regression*

Table A summarises the results from an assessment using multinomial logistic regression of the influence of prognostic factors on mortality, both for breast cancer specifically, and from other causes, for the whole sample. In the multiple regression setting, all factors contributed to the prediction of breast cancer-specific mortality. The strongest predictors were stage and grade, but there were also associations with individual categories of age, comorbidity and screen detection. For other-cause mortality, age and comorbidity were the strongest predictors, but stage also contributed to prediction of other-cause mortality, albeit to a much lesser degree than for breast cancer mortality. This model has some limitations. It does not consider potential interactions between prognostic factors, nor the dependence between them, which are considered further in the main text. The component of the model that predicted breast cancer mortality vs survival formed the basis for the development of prognostic scores, explained in detail below.

*2. Interactions between prognostic factors*

Table B (Supplementary). Tests for interactions between prognostic risks in logistic regression of breast cancer specific mortality

| Model | ꭓ^2^ | df | p | Pseudo-Adj R^2^ | ΔR^2^ | BIC/ Δ_BIC_ | Strength |
| --- | --- | --- | --- | --- | --- | --- | --- |
| Base Model 1: Stage | 2307.6 | 3 | <.001 | .293 |  | 5569.37 |  |
| +Grade | 237.69 | 4 | <.001 | .323 | .029 | 199.56 | +V. Strong |
| +Age Category | 74.23 | 7 | <.001 | .304 | .008 | 7.50 | +Strong |
| +Screen Detected | 52.60 | 1 | <.001 | .300 | .006 | 43.06 | +V. Strong |
| +Comorbidity | 33.44 | 2 | <.001 | .297 | .004 | 14.37 | +V. Strong |
| Base Model 2: All Main Effects | 2700.3 | 17 | <.001 | .340 |  | 5310.09 |  |
| -Grade | 238.83 | 4 | <.001 | .310 | -.029 | -200.69 | -V.Strong |
| -Age Category | 84.34 | 7 | <.001 | .331 | -.009 | -17.60 | -V.Strong |
| -Screen Detected | 37.59 | 1 | <.001 | .335 | -.005 | -28.06 | -V.Strong |
| -Comorbidity | 26.17 | 2 | <.001 | .337 | -.003 | -7.10 | -Strong |
| +Stage by Age | 36.42 | 21 | <.02 | .339 | -.001 | -163.80 | -V.Strong |
| +Stage by Screen Detected | 3.759 | 3 | n.s. | .340 | .000 | -24.84 | -V.Strong |
| +Stage by Comorbidity | 11.70 | 6 | n.s. | .340 | .000 | -45.50 | -V.Strong |
| +Age by Screen Detected | 9.51 | 7 | n.s. | .339 | -.001 | -57.23 | -V.Strong |
| +Age by Comorbidity | 21.60 | 14 | n.s. | .339 | -.001 | -111.9 | -V. Strong |
| +Scr. Detected by Comorbidity | 2.04 | 2 | n.s. | .340 | .000 | -17.02 | -V.Strong |
| +Stage by Grade | 14.32 | 9 | n.s. | .339 | .000 | -71.478 | -V.Strong |
| +Age Category by Grade | 30.20 | 21 | n.s. | .338 | -.002 | -170.00 | -V.Strong |
| +Screen Detected by Grade* | 19.98 | 3 | <.001 | .341 | .002 | -8.63 | -Strong |
| +Comorbidity by Grade | 7.03 | 6 | n.s. | .339 | -.001 | -50.17 | -V.Strong |

*To aid interpretation of differences in Bayesian Information Criteria (BICs), verbal descriptions of evidence against

a higher BIC were developed by R.E. Kass and A.E. Rafferty (1995) “Bayes Factors”, Journal of the American Statistical Association, 90(430):773:795. A BIC of 2 or more is considered “positive” evidence, 6 or more “Strong” evidence and 10 or more “Very Strong” evidence.

We explored whether the model predicting breast cancer specific mortality from prognostic factors was improved by including interactions between those factors (Table B). The base logistic regression model was a univariate model consisting the strongest prognostic factor of breast cancer specific mortality, the stage of the disease at diagnosis. Main effect marginal associations of the other prognostic factors were then assessed by adding them one at a time to the base model. All prognostic factors significantly improved the fit of the logistic regression when added to the base model. The adjusted pseudo-R^2^ improved, and the Bayesian Information Criteria (BICs) indicated that this improvement was not due to overfitting. Similarly, when the base model included all the main effects of the set of prognostic factors, partial associations were assessed by dropping one prognostic factor at a time. This significantly reduced the fit of the model in every case; reduced the adjusted pseudo-R^2^; and BICs provided strong or very strong evidence that there was no overfitting of the main effect model. In the case of interaction terms, only one produced a significant improvement in model fit according to the likelihood ratio ꭓ^2^ test, but this improvement was due to overfitting according to the BIC, and also according to the less conservative Akaike Information Criterion (not shown). The main effect (additive) logistic regression model therefore represents the optimum prognostic model.

*3. Observed mortality rates as a function of SES and rurality*

Table C (Supplementary). Observed five-year breast cancer-specific and other-cause mortality for women with breast cancer as a function of SES and rurality of residence. Confidence intervals are unadjusted.

| SES | Rurality | Alive | Breast Cancer  Death | | Other-Cause  Death | | | N  (14,165) | % of cohort in stratum |
| --- | --- | --- | --- | --- | --- | --- | --- | --- | --- |
|  |  | % | % | Conf. Interval | % | | Conf. Interval |  |  |
| Highest | City | 92.36 | 5.78 | (4.98-6.70) | 1.86 | (1.42-2.42) | | 2,853 | 20.14 |
|  | Inner | 94.80 | 3.72 | (2.01-6.77) | 1.49 | (0.06-3.89) | | 269 | 1.90 |
|  | Outer | 84.62 | 15.38 | (3.87-45.1) | 0.00 | - | | 13 | 0.09 |
| Higher | City | 90.79 | 7.33 | (6.35-8.44) | 1.88 | (1.41-2.51) | | 2,388 | 16.86 |
|  | Inner | 92.68 | 5.89 | (4.13-8.35) | 1.42 | (0.68-2.95) | | 492 | 3.47 |
|  | Outer | 89.53 | 8.14 | (3.93-16.1) | 2.33 | (0.58-8.82) | | 86 | 0.61 |
| Middle | City | 88.68 | 8.75 | (7.57-10.1) | 2.57 | (1.96-3.38) | | 1,943 | 13.72 |
|  | Inner | 90.91 | 6.89 | (5.22-9.05) | 2.22 | (1.33-3.62) | | 682 | 4.81 |
|  | Outer | 91.30 | 7.07 | (4.15-11.8) | 1.63 | (0.53-4.93) | | 184 | 1.30 |
| Lower | City | 87.37 | 9.47 | (8.12-11.0) | 3.16 | (2.40-4.14) | | 1,584 | 11.18 |
|  | Inner | 88.81 | 7.96 | (6.33-9.96) | 3.23 | (2.24-4.64) | | 867 | 6.12 |
|  | Outer | 87.84 | 11.71 | (8.10-16.7) | 0.45 | (0.06-3.13) | | 222 | 1.57 |
| Lowest | City | 86.05 | 10.53 | (9.10-12.2) | 3.42 | (2.62-4.45) | | 1,548 | 10.93 |
|  | Inner | 87.42 | 9.06 | (7.25-11.3) | 3.52 | (2.44-5.05) | | 795 | 5.61 |
|  | Outer | 82.83 | 12.55 | (8.92-17.4) | 5.02 | (2.87-8.63) | | 239 | 1.69 |

*4. Pairwise associations between prognostic factors*

Nominal associations between prognostic factors were identified using Cramer’s V ^1^ (Table D).

*Table D (Supplementary)*. Nominal associations between prognostic factors

|  |  |  |  |  |  |
| --- | --- | --- | --- | --- | --- |
|  | Cramer’s V | | | | |
| Stage | * |  |  |  |  |
| Grade | 0.253 | * |  |  |  |
| Comorbidity | 0.071 | 0.037 | * |  |  |
| Screen Detected | 0.282 | 0.191 | 0.066 | * |  |
| Age Category | 0.077 | 0.087 | 0.080 | 0.372 | * |
|  | Stage | Grade | Comorbidity | Screen  Detected | Age Category |

*5. Prognostic scores from the reference group and the whole cohort (adjusted).*

As explained in the main article, prognostic scores may be subject to confounding if the “exposure” factors (in this case socio-demographic factors) influence both the prognostic profiles within levels of exposure as well as the outcome (in this case, breast cancer-specific mortality). This means that prognostic scores should be calculated based on the mortality risk in a reference group, to exclude the effect of factors that come into play post-diagnosis, such as treatment. For example, patients from disadvantaged areas may be more likely to be diagnosed at a later stage. However, if they do not receive comparable treatment, given the same stage at diagnosis, this will contaminate the estimate of the effect of being diagnosed at a later stage. Thus, in principle, prognostic scores should be derived from the reference group, and then applied to the other groups. However, Arbogast and Ray (2011 – reference in main text) have shown that prognostic scores can be derived from the whole group if the correlation between the exposure factors and prognostic factors is weak, which is true of our sample. Using the whole group has the advantage that scores are more precise.

As a check, we compared prognostic scores constructed from the reference group — highest SES city dwellers — to those obtained from the entire cohort. Briefly, we used multiple logistic regression to construct models from the reference group and the whole group using the prognostic factors listed in Table A above. Only the logistic regression model that discriminated those who were alive at five years follow-up from those who died due to their breast cancer was considered. We then used linear regression to relate the two sets of scores and adjusted the regression slope and intercept of the whole group prognostic scores to match those of the reference group (excluding some scores that exhibited extrapolation failure from the reference group). As shown in Figure A, the resulting average prognostic risks were extremely similar, although the reference group prognostic risks were noisier as they were based on a smaller sample size. Importantly, there was no evidence that the use of the entire cohort to construct prognostic scores introduced bias. That means that the scores based on the entire sample could be used to estimate the degree to which differential prognosis mediates survival disparities associated with SES and rurality, as explained in the main text.


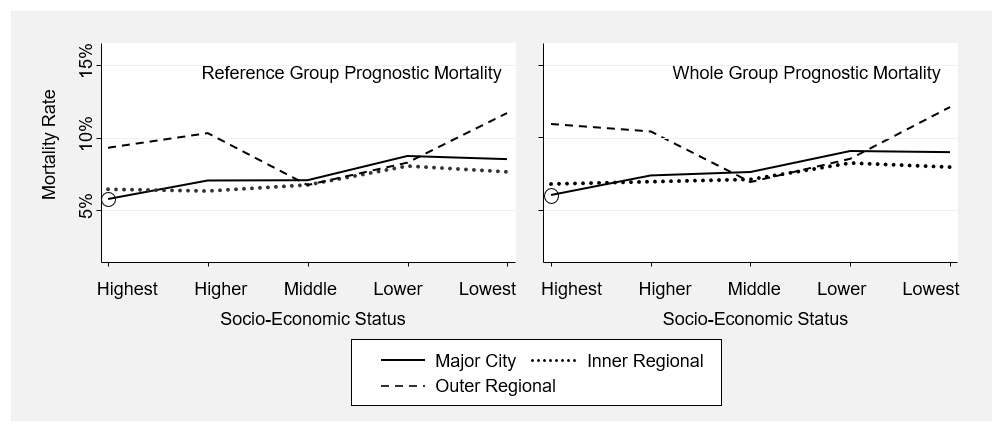


Figure A (Supplementary). Predicted mortality based on prognostic scores estimated from the reference group (circled) [left panel] and the whole group [right panel].

6. Associations between individual prognostic risks, SES and Rurality

Table E shows the distribution of individual prognostic factors broken down by both SES and rurality. Note the higher percentage of Grade 4 breast cancers in the lower SES areas, the higher percentage of Stage 1 cancers in the higher SES areas and the higher frequency of reported comorbidities in the lower SES areas.

Table E (Supplementary). Percentage distribution of prognostic categories by Rurality and SES.

| Rurality | Major City | | | | | Inner Regional | | | | | Outer Regional | | | | |
| --- | --- | --- | --- | --- | --- | --- | --- | --- | --- | --- | --- | --- | --- | --- | --- |
| SES | *Lowest* | *Lower* | *Middle* | *Higher* | *Highest* | *Lowest* | *Lower* | *Middle* | *Higher* | *Highest* | *Lowest* | *Lower* | *Middle* | *Higher* | *Highest* |
| n | 1548 | 1584 | 1943 | 2388 | 2853 | 795 | 867 | 682 | 492 | 269 | 239 | 222 | 184 | 86 | 13 |
| Stage |  |  |  |  |  |  |  |  |  |  |  |  |  |  |  |
| Stage 1 | 43.35 | 44.82 | 47.76 | 47.78 | 53.49 | 47.30 | 48.21 | 51.17 | 51.63 | 46.10 | 41.00 | 40.99 | 54.89 | 36.05 | 38.46 |
| Stage 2 | 36.76 | 35.23 | 34.69 | 35.09 | 32.88 | 34.34 | 32.87 | 30.06 | 31.71 | 36.43 | 35.98 | 39.64 | 28.26 | 38.37 | 23.08 |
| Stage 3 | 11.89 | 11.62 | 11.32 | 11.31 | 9.11 | 11.70 | 11.42 | 13.34 | 10.77 | 13.01 | 10.88 | 13.06 | 10.87 | 18.60 | 30.77 |
| Stage 4 | 8.01 | 8.33 | 6.23 | 5.82 | 4.52 | 6.67 | 7.50 | 5.43 | 5.89 | 4.46 | 12.13 | 6.31 | 5.98 | 6.98 | 7.69 |
| Grade |  |  |  |  |  |  |  |  |  |  |  |  |  |  |  |
| Low | 15.96 | 16.10 | 16.21 | 16.82 | 17.60 | 16.60 | 16.61 | 17.01 | 17.68 | 16.73 | 13.39 | 13.51 | 17.93 | 9.30 | 7.69 |
| Intermediate | 44.96 | 43.69 | 43.18 | 45.06 | 46.09 | 44.15 | 43.14 | 46.48 | 47.15 | 41.26 | 37.66 | 42.79 | 40.22 | 39.53 | 46.15 |
| High | 33.40 | 34.91 | 35.62 | 34.46 | 32.84 | 34.34 | 34.83 | 30.65 | 30.28 | 37.55 | 40.59 | 37.84 | 36.96 | 45.35 | 38.46 |
| Unknown | 5.68 | 5.30 | 4.99 | 3.56 | 3.47 | 4.91 | 5.42 | 5.87 | 4.88 | 4.46 | 8.37 | 5.86 | 4.89 | 5.81 | 7.69 |
| Comorbidity |  |  |  |  |  |  |  |  |  |  |  |  |  |  |  |
| None | 79.33 | 80.18 | 85.02 | 85.85 | 87.49 | 79.25 | 79.12 | 81.23 | 80.69 | 85.13 | 71.13 | 74.77 | 70.65 | 77.91 | 76.92 |
| No Admission | 16.99 | 16.16 | 13.48 | 12.69 | 11.25 | 18.36 | 18.80 | 17.01 | 17.68 | 13.38 | 25.94 | 23.87 | 26.09 | 19.77 | 23.08 |
| Present | 3.68 | 3.66 | 1.49 | 1.47 | 1.26 | 2.39 | 2.08 | 1.76 | 1.63 | 1.49 | 2.93 | 1.35 | 3.26 | 2.33 | 0.00 |
| Screen Detect. |  |  |  |  |  |  |  |  |  |  |  |  |  |  |  |
| Yes | 32.95 | 33.59 | 31.29 | 31.87 | 32.60 | 35.97 | 38.18 | 36.07 | 35.77 | 38.29 | 35.98 | 38.29 | 37.50 | 32.56 | 38.46 |
| No | 67.05 | 66.41 | 68.71 | 68.13 | 67.40 | 64.03 | 61.82 | 63.93 | 64.23 | 61.71 | 64.02 | 61.71 | 62.50 | 67.44 | 61.54 |
| Age Category |  |  |  |  |  |  |  |  |  |  |  |  |  |  |  |
| 16-45 | 13.82 | 16.48 | 18.01 | 18.59 | 17.67 | 12.70 | 13.38 | 15.10 | 11.79 | 13.01 | 11.30 | 10.81 | 14.67 | 11.63 | 15.38 |
| 46-50 | 12.53 | 13.89 | 13.48 | 14.82 | 15.21 | 11.82 | 9.57 | 14.08 | 13.82 | 15.24 | 14.23 | 10.36 | 16.85 | 13.95 | 7.69 |
| 51-55 | 13.24 | 12.63 | 14.31 | 13.82 | 15.63 | 11.45 | 14.30 | 13.20 | 18.90 | 16.36 | 12.13 | 10.81 | 15.76 | 18.60 | 15.38 |
| 56-60 | 15.18 | 15.59 | 14.51 | 14.20 | 15.46 | 13.08 | 14.30 | 14.37 | 12.80 | 17.10 | 13.81 | 18.02 | 14.67 | 11.63 | 30.77 |
| 61-65 | 15.50 | 14.77 | 15.65 | 14.57 | 14.79 | 16.60 | 17.30 | 18.77 | 17.28 | 19.33 | 14.64 | 20.72 | 11.41 | 12.79 | 7.69 |
| 66-70 | 13.76 | 14.08 | 11.27 | 12.23 | 11.53 | 13.96 | 16.96 | 11.44 | 16.26 | 11.15 | 12.55 | 15.32 | 14.13 | 11.63 | 15.38 |
| 71-75 | 10.01 | 8.46 | 8.59 | 8.46 | 6.94 | 14.34 | 9.34 | 9.68 | 7.11 | 5.95 | 15.48 | 9.01 | 8.15 | 10.47 | 7.69 |
| >75 | 5.94 | 4.10 | 4.17 | 3.31 | 2.77 | 6.04 | 4.84 | 3.37 | 2.03 | 1.86 | 5.86 | 4.95 | 4.35 | 9.30 | 0.00 |
